# Systems redox biology in health and disease

**DOI:** 10.17179/excli2022-4793

**Published:** 2022-03-21

**Authors:** Martin Feelisch, Miriam M. Cortese-Krott, Jérôme Santolini, Stephen A. Wootton, Alan A. Jackson

**Affiliations:** 1 Clinical and Experimental Sciences, Faculty of Medicine, University of Southampton and NIHR Biomedical Research Center, University Hospital Southampton, NHS Foundation Trust, Tremona Road, Southampton, SO16 6YD, UK; 2 Myocardial Infarction Research Laboratory, Department of Cardiology, Pulmonology and Angiology, Medical Faculty, Heinrich Heine University of Düsseldorf, Moorenstr. 5, D-40225 Düsseldorf, Germany; 3 Institute for Integrative Biology of the Cell (I2BC), CEA, CNRS, Univ Paris-Sud, Université Paris-Saclay, F-91198, Gif-sur-Yvette Cedex, France; 4 Institute of Human Nutrition, University of Southampton and University Hospital Southampton, Tremona Road, Southampton, SO16 6YD, UK

**Keywords:** integrated physiology, systems medicine, oxidative stress, hydrogen sulfide, redox signaling, nutrition

## Abstract

Living organisms need to be able to cope with environmental challenges and other stressors and mount adequate responses that are as varied as the spectrum of those challenges. Understanding how the multi-layered biological stress responses become integrated across and between different levels of organization within an organism can provide a different perspective on the nature and inter-relationship of complex systems in health and disease. We here compare two concepts which have been very influential in stress research: Selye's '*General Adaptation Syndrome*' and Sies's '*Oxidative Stress*' paradigm. We show that both can be embraced within a more general framework of 'change and response'. The '*Reactive Species Interactome*' allows each of these to be considered as distinct but complementary aspects of the same system, representative of roles at different levels of organization within a functional hierarchy. The versatile chemistry of sulfur - exemplified by hydrogen sulfide, glutathione and proteinous cysteine thiols - enriched by its interactions with reactive oxygen, nitrogen and sulfur species, would seem to sit at the heart of the 'Redox Code' and underpin the ability of complex organisms to cope with stress.

## Introduction


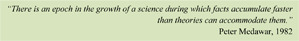


All life forms, from single cells to complex organisms such as humans, are challenged by the environment in which they live. These challenges vary across a wide spectrum, from the mildest perturbations at one end to the most severe stressors at the other. The challenges vary not only in intensity but also in nature and duration, and the responses may be generic or highly specific. A common feature of all organisms that allows them to survive and maintain health is the ability to mount a response that is similarly varied, tends to be proportionate and appears fit for purpose. While milder perturbations are accommodated locally, for the more severe stressors the response is escalated through higher levels of organization to a fully integrated whole-body response. The interaction of the processes coming into play as the magnitude of the response increases is understood, in part, at individual levels of biological organization. But how these horizontal and vertical responses are co-ordinated in time and space remains to be defined. These layered responses exhibit both quantitative and qualitative characteristics as one moves through the escalation at different levels of organization; in general, there is an increase in the capacity to accommodate stressors with engagement of each successive higher level of organization. Thus, a general feature of involving successively higher levels is a greater capacity to cope with (or buffer) the magnitude and effect of a challenge. In other words, the progressive engagement or recruitment at higher levels enables more severe stresses to be accommodated without compromising overall function. 

The essence of the term '*stress*' implies an exposure that challenges the ability of a cell/organism to cope. The ability to respond to more severe stress by calling on greater buffering capacity through the recruitment of higher levels of organization implies and requires integration within and between the different levels of organization. 

In earlier perspectives (Cortese-Krott et al., 2017[8]; Santolini et al., 2019[54]) we have explored the extent to which the response to stressors of various kinds could have emerged as an integral feature of the evolutionary process. We have argued that life forms selectively acquired improved coping capabilities in response to changes in the environment, and to an extent this may account for biological variability. The progressive acquisition of increasingly sophisticated coping capabilities may present itself as an *'emergent property'*, with each successive experience being layered onto previously acquired characteristics. Hence, the complex multi-layered capabilities identified within humans (and other large organisms) reflect the progressively acquired layers that were selected throughout evolution as 'fit for purpose' and enabled their survival over long periods of time. 

**Figure 1:**
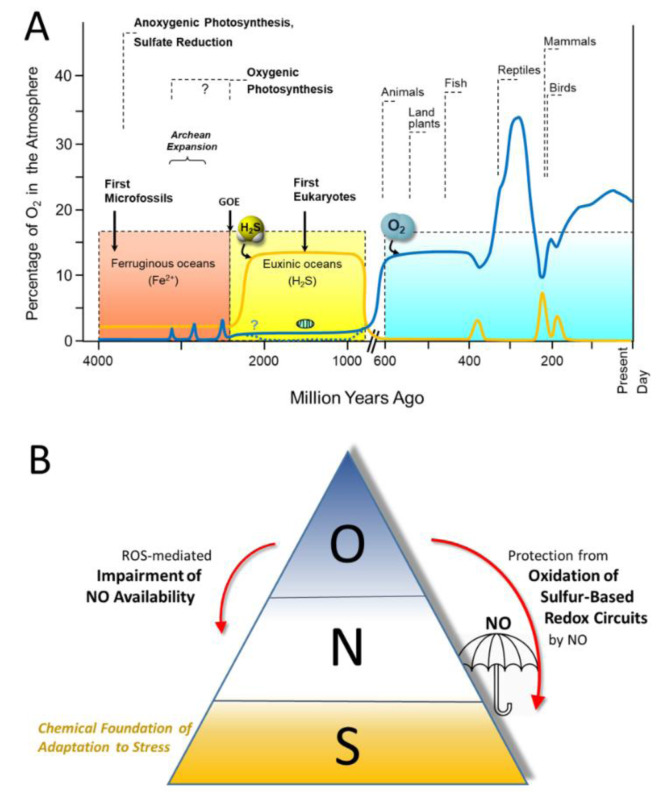
A. Evolution of sulfur and oxygen metabolism. At the onset of Life, approximately 3.8 billion years ago, the environment was devoid of O_2_. The first eukaryotes appearing in the oceans evolved under euxinic (anoxic/sulfidic) conditions, using sulfur-containing molecules as their energy source and generating reactive sulfur species in the process. With the emergence of cyanobacteria capable of splitting water by harnessing sunlight (oxygenic photosynthesis) large amounts of O_2_ were generated, leading to gradual oxidation of dissolved hydrogen sulfide (H_2_S) and reduced iron (Fe^2+^) during the *Great Oxidation Event* (GOE). Multicellular life forms appeared around the time when O_2_ started to accumulate in the atmosphere (~600 million years ago); with the additional production by plants, atmospheric O_2_ concentrations rose dramatically*.* Blue line: atmospheric oxygen concentrations; yellow line: concentration of sulfide dissolved in the oceans. Modified from (Olson and Straub, 2016; Cortese-Krott et al., 2017). B. Presumed order of utilization of Sulfur (S), Nitrogen (N) and Oxygen (O) based redox chemistry governing stress signaling, survival and resilience during the evolutionary development of Life. In contemporary mammalian life forms, stress signaling and adaptation is based on the intricate interaction of sulfur-based redox modules, the function of which is susceptible to perturbation or damage by reactive oxygen species (ROS). Adequate formation of nitric oxide (NO) is important for the protection of the sulfur-based circuitry that is critical to resilience and survival of mammalian organisms, and enhanced ROS production also poses a threat to the bioavailability of NO (from Cumpstey et al., 2021).

We and others have argued that as life emerged in a sulfur-rich environment (Figure 1(Fig. 1); References in Figure 1: Cortese-Krott et al., 2017[8]; Cumpstey et al., 2021[10]; Olson and Straub, 2016[49]) the ability to effectively handle sulfur must have played a fundamental role in the development of these survival attributes (Olson and Straub, 2016[49]; Cortese-Krott et al., 2017[8]). As subsequent response and survival capabilities emerged, they were layered onto existing capabilities, allowing ever more complex interactions to emerge. We have used these ideas to develop the concepts of the '*Reactive Species Interactome' *(RSI) (Cortese-Krott et al., 2017[8], 2020[9]) and the '*Redox Interactome*' (Santolini et al., 2019[54]). 

We applied these paradigms to explain the complex nature of physiological adaptations that occur in individuals subjected to combined environmental and metabolic stress in health (Cumpstey et al., 2019[11]) as well as in disease settings; with regard to the latter, we explored the redox-related perturbations in physiology in survivors and non-survivors of critical illness (McKenna et al., 2021[41]), in the context of achieving redox balance during viral infection with SARS-CoV2 (Cumpstey et al., 2021[10]), and in relation to alterations in the pulmonary and systemic redox landscape that underpin the reduction in inflammation and improvement in quality of life of asthma patients enrolled in a structured exercise training program (Freeman et al., 2021[20]). 

Here, we provide a further perspective that explores how the RSI may allow to understand how these complex, emergent properties become integrated while providing a different perspective on the nature and inter-relationship of health and ill-health. For this, consideration is given to a single-layered capability, which provides a '*Framework of change and response*'. 

Moreover, we consider how this framework offers opportunities that are of practical relevance. We begin with two concepts that have been very influential in the development of ideas, the “*General Adaptation Syndrome*” of Selye, and the “*Oxidative Stress*” paradigm developed by Sies. Each of these explores how an organism might respond to, or cope with, challenges or 'stress'. We consider that both need to be embraced within any general model. However, as each is illustrative of responses at very different levels of organization, together they invite consideration of how they might relate to each other, thereby exemplifying the inter-relationships amongst the different levels of organization. Selye offered a clinicians' perspective of how aspects of the stress response are integrated as parts of a whole-body response, primarily through hormone-dependent processes. Sies offers a mechanistic perspective based upon our understanding of cellular biochemistry. The RSI allows both of these to be considered as distinct yet complementary aspects of the same system representative of roles at different levels of organization within a functional hierarchy.

## Metamorphosis of the Stress Concept – From Whole-body Response via Oxidative Damage to Redox Regulation

***Selye.*** The stress concept (initially known as '*general adaptation syndrome*') was introduced to biology by Hans Selye more than 80 years ago to describe the response of the body when challenged by a stressful stimulus or a noxious substance (Selye, 1936[58]). During his medical studies Selye had observed that patients suffering from very different diseases often had common non-specific complaints (the '*syndrome of feeling sick*'). He later developed these concepts further, largely based upon experimental studies in experimental animals.

The term '*stress*' was borrowed from physics where it referred to the action of a force on inanimate material and the resistance to counter that force. By introducing it to the biological literature Selye repurposed it to describe a '*non-specific response of the body to any demand'*. He divided the overall response into three phases: the acute alarm phase, the attempt of the body to maintain homeostasis by resisting the change, and the eventual stage of exhaustion. These ideas became widely recognized and considered to represent an important conceptual insight. Although Selye emphasized that stress was a necessary and useful physiological response, it soon assumed a negative connotation, especially in non-scientific circles and when discussed in a psychosocial context. Implicit in the articulation was the notion of a graded response to challenge; for those stressors that did not lead to death there was a learnt ability that offered resilience to future exposure to a similar stressor. Following Levi's distinction between “positive stress” and “negative stress” (Levi, 1971[36]), Selye introduced the terms “eustress” and “distress” to distinguish stress responses that were initiated by positive emotions from those triggered by negative, unpleasant causes (Selye, 1974[59]). For Selye, and in keeping with his time, the emphasis was on the whole-body responses to stressors mediated through the hypothalamic-pituitary-adrenal (HPA) axis and known to be dependent upon neuronal and endocrine communications.

***Sies.*** Similar considerations about the quality of the response to different degrees of stress or different stressors are currently explored in relation to research on oxidative stress (Niki, 2016[46]; Sies, 2015[63], 2018[61]). In the introduction to Helmut Sies's eponymous monograph on oxidative stress it was defined as a “disturbance in the pro-oxidant/anti-oxidant system in favor of the former” (Sies, 1985[62]). These are clearly responses at the molecular, sub-cellular and cellular levels. Inflammatory processes provide an illustrative example. The '*oxidative burst*' (during which reactive oxygen species (ROS) are produced in large quantities) is a feature of macrophages that have been activated by invasive pathogens and facilitates their killing (Flohe et al., 1985[18]). Compared with the resting state there is relative overproduction of ROS, which exceeds the usual capacity for their removal with a consequent loss in the dynamic equilibrium between their rates of formation (as byproducts of aerobic metabolism or by targeted production via NADPH oxidases, NOXs) and removal (by superoxide dismutase, catalase, peroxidases and peroxyredoxins). A local imbalance can lead to ***Oxidative Stress*** and structural damage, not only at the cellular level but also in surrounding tissue. 

Conceptually, this offers a coherence that makes the *oxidative stress* idea easy to grasp. It has gained popularity across a range of biological disciplines and has been greeted by the scientific community with an even greater level of enthusiasm than Selye's original stress concept. Oxidative stress has also changed our perception of disease processes. It has made it possible to identify and classify '*redox diseases'* (including atherosclerosis, diabetes, neurodegenerative diseases, and chronic inflammation) as conditions that involve oxidative stress (Sies, 2015[63]). An updated concept considers the need for a clearer differentiation between “purposeful” and undesirable ROS formation/degradation by way of distinguishing between “oxidative eustress” and “oxidative distress” (Sies, 2020[60]). Unfortunately, oversimplification has encouraged the erroneous impression that ROS (and other oxidants) are always “bad” and largely associated with pathological processes beyond the destruction of potentially pathogenic bacteria. As a corollary, antioxidants are often considered to be “good” and likely to have therapeutic benefit in treating oxidative stress related disorders. 

Clinical trials have not provided support for this overly simplistic perspective, and more often than not the purported therapeutic value of antioxidants has not been realized in practice (Forman and Zhang, 2021[19]). Thus, although the concept has value it does not provide a sufficiently adequate understanding of how responses might operate at the level of the whole body (Jones, 2006[32]; Sies, 2015[63], 2018[61]). The editors of another recent monograph on the subject suggest that systems medicine and network pharmacology-based mechanistic approaches may soon replace the notion of 'oxidative stress'-related diseases and the concept of antioxidants altogether (Schmidt et al., 2021[55]).

### From ROS via other Reactive Species to Redox Regulation

In the first two decades following the introduction of the 'oxidative stress' concept much effort was devoted to understand how structural and functional cellular constituents such as lipids, proteins and DNA are oxidatively damaged by excess ROS. With time, it became increasingly clear that ROS were not simply damage-inflicting by-products of inflammatory processes, but also potent signaling molecules in their own right, making them important elements of a wider network of cellular communication (Jones, 2006[32]; Sies, 2015[63]). Nowadays, ROS - along with reactive nitrogen species (RNS) and the more recently introduced reactive sulfur species (RSS) - are thought to be integral to electron transfer processes and cellular stress responses. This development facilitated the birth of “redox biology”, a new area of research focusing on redox signaling and regulation (Sies, 2015[63]).

Although some contemporary literature still considers that “reactive species lack a well-defined chemistry”, it is now evident that each chemical entity has a unique bioactivity and interacts with selected biological targets in a highly specific manner. In the context of redox signaling, compounds with a sulfhydryl (-SH) group, i.e. thiols, are often the target of reactive species not only in the process of detoxification (e.g. via coupling to glutathione and other small-molecular-weight thiols) but also in their “role” as redox switches. Examples include reactive thiols in regulatory proteins, leading to short (seconds to minutes), medium (minutes to hours), or long-term (days, years, generations) effects. These effects may involve membrane or intracellular signaling proteins and induce alterations in protein structure and/or activity, modulation of protein-protein interactions or gene expression, and even induce heritable epigenetic/genetic modifications. 

The more recent introduction of the concept of the '***Redox Code***' (Jones and Sies, 2015[33]) has been used with the meaning of a '*set of principles*'. While the '*Genetic Code'* contains the blueprint for the production of proteins and regulatory elements necessary for cell survival, the '*Redox Code*' contains the information for cellular redox responses; specifically, enzymatically-driven, highly regulated responses that are coupled to the availability of reducing equivalents (NADPH, NADH) and the cellular energetic status. This model was a major step forward in helping to redefine oxidative stress as a state associated with dysregulated redox signaling (Sies, 2018[61]). Even so, it focused almost exclusively on the enzymatic production of superoxide (O_2_^·-^), hydrogen peroxide (H_2_O_2_) and derived species. However, ROS themselves have the potential to engage in complex chemical interactions with a range of other small molecules such as RNS and RSS, even before they interact with more complex molecules such as DNA, protein or lipid targets. For example, the formation of superoxide impairs the bioavailability of nitric oxide (NO) (Gryglewski et al., 1986[25]), which can contribute to endothelial dysfunction and lead to the generation of the secondary RNS, peroxynitrite (ONOO^-^) (Beckman et al., 1990[4]; Beckman and Koppenol, 1996[5]). 

### Reactive Species interactions and the Redox Interactome

**Figure 2:**
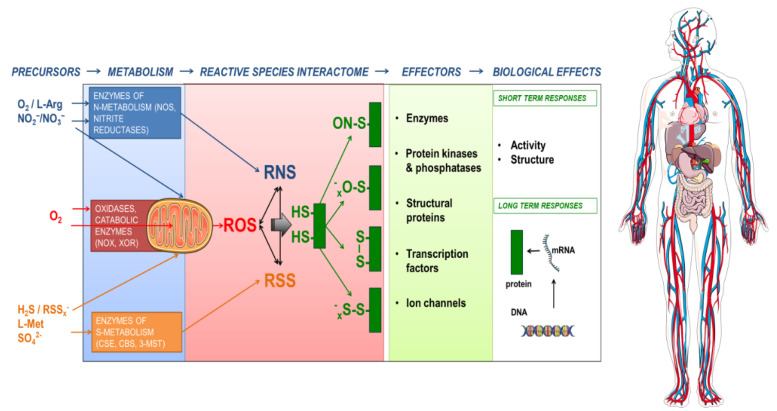
The Reactive Species Interactome in action. The constituting elements of the reactive species interactome (RSI) are reactive oxygen species (ROS), reactive nitrogen species (RNS) and reactive sulfur species (RSS) together with thiols as their biological targets. Their chemical interactions lead to formation of a multitude of products with different reactivities, stabilities, half-lives and physicochemical properties, covering a wide range of maximal travel distances. Precursors of the RSI are organic and inorganic substances and cofactors, which are transformed by mitochondrial or cytoplasmic enzymes. A common target of the RSI are cysteine thiols in proteins, acting as redox switches, able to fine-tune activity of signaling molecules and leading to short-term responses or long-term adaptation (by redox switches or by modifying gene expression/regulation). The lymphatic system and the circulation serve as trade routes for exchange and communication. Circulating thiols and longer-lasting products of the RSI (e.g. nitrite, polysulfides) also serve as local and systemic heterocellular communication entities. Precursor availability, metabolism, signaling and mitochondrial function depend on the nutritional and physiological status of the organism and affect the RSI (modified from Cortese-Krott et al., 2017)

**Figure 3:**
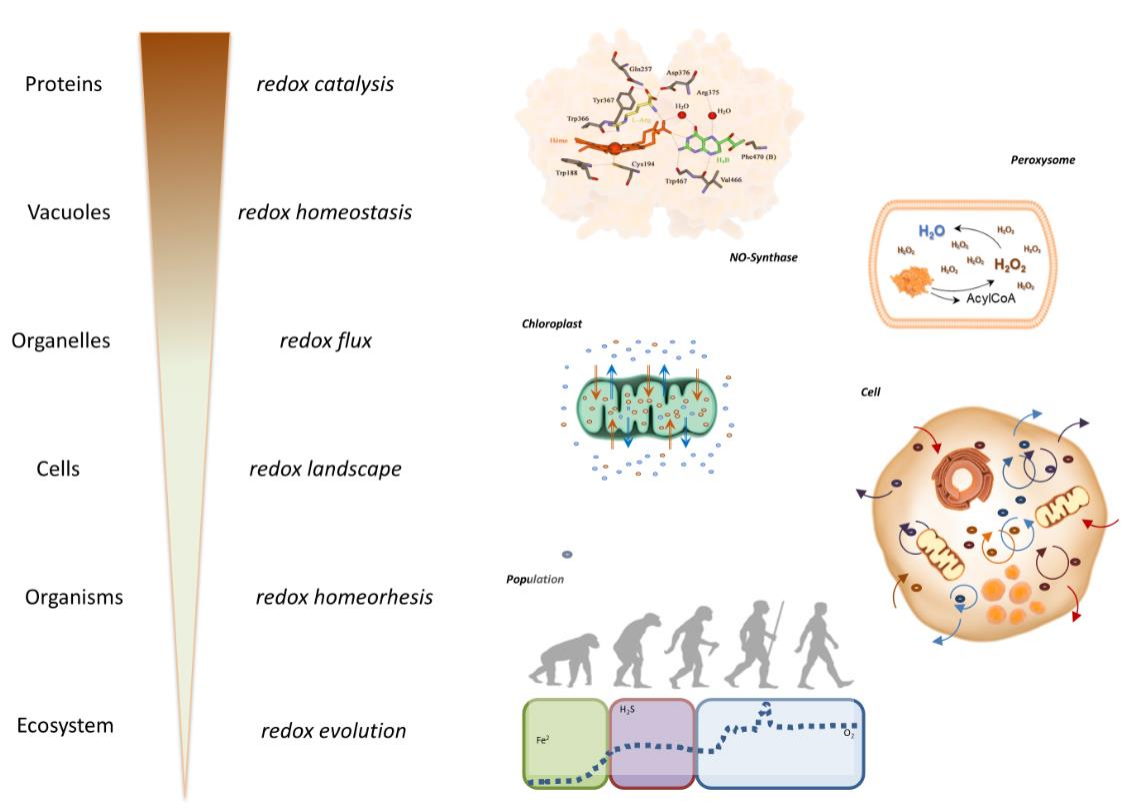
Increasing Complexity: Stabilizing Redox Machineries. The increasing complexity of redox machineries is depicted here as an example of multilayered regulation and increasing complexity of stress response mechanisms and the compartmentalization and synchronization of different redox biochemistries. These include the catalytic pocket of a protein (here, nitric oxide synthase), the isolated oxidative chemistry of a peroxisome, the build-up of transmembrane electrochemical gradients in a chloroplast, the intracellular coupling of redox processes within a cell, and the variations in redox environments coupled to organismal evolution (modified from Santolini et al., 2019).

In order to be able to better understand these complex interactions we introduced the concept of the ***Reactive Species Interactome*** (RSI) (Figure 2(Fig. 2); Reference in Figure 2: Cortese-Krott et al., 2017[8]). It is possible to consider how the concept of the ***Redox Code*** interplays with the serendipity of the 'Chemistry of Life' and hence to assess how, through natural selection, the progressive acquisition of structure and order enables the emergence of the complex capabilities as a fundamental feature that enables Life to cope with a range of environmental stressors (Cortese-Krott et al., 2017[8]). Thus, it has been possible to characterize the set of chemical interactions of reactive species (i.e. RSS, RNS and ROS) among themselves and with other biological entities. These biochemical reactions take place in both the intracellular and extracellular space, providing a mechanism through which cells can sense their environment and adjust their metabolic machinery to enable, promote or support the dynamic equilibrium. Since the RSI is embedded within a wider redox network, which connects the internal redox system of a cell or an organism to its environment, there are likely implications of this for the redox landscape in which the system operates. These considerations have been captured in the form of the ***Redox Interactome ***(Santolini et al., 2019[54]) (Figure 3(Fig. 3); Reference in Figure 3: Santolini et al., 2019[54]). 

Mechanistically and in the form of a universal language, redox chemistry provides a bridge between living processes and the Earth's major chemical cycles, integrating biosphere, lithosphere, hydrosphere and atmosphere into dynamic S, N, C and O cycles. Redox chemistry is by definition also a source of infinite variety: coupled chemical reactions produce new compounds that in turn engage in new reactions that produce different molecules, extending the number and nature of structures and interactions while exploring new chemical spaces. 

**Figure 4:**
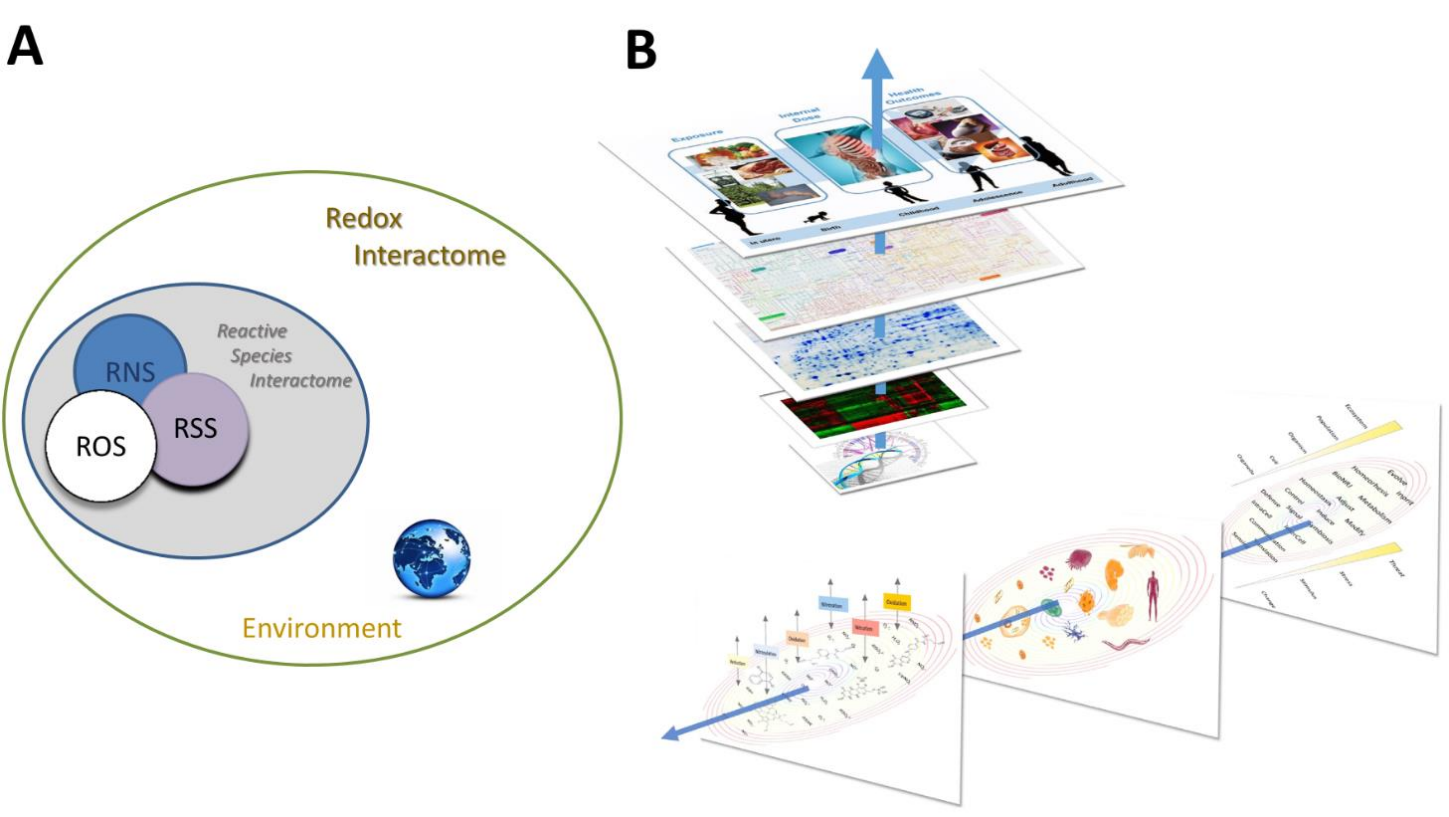
The Redox Interactome and the Redox Spine connecting all layers of regulation. (A) Redox chemistry pervades all metabolic and functional reactions. The simultaneous superimposition of multiple activities of the RSI remains interconnected through a common *Redox Interactome, *the nature of which is determined by the physicochemical properties of the environment. (B) Upper part: The ‚redox spine' (blue arrow) connects the different layers of biological organization within an organism. Lower part: From right to left the panels represent 1. Evolution and diversification of redox function, from sensing to adaptation. 2. The complexification of redox structure as a function of time, from biomolecules to organisms. 3. Multiplicity and evolution of redox agents (the arrows reflect the various types of redox chemistry as a function of time/environment) (modified from Santolini et al., 2019)

Thus, originating from a small number of starting molecules this self-organizing (and self-perpetuating) chemistry led to increasingly complex structures, thereby enabling increasingly complicated functions. Over time, the evolution of the geochemical environment and the diversification of life forms offered the possibility to explore new chemical combinations within highly compartmentalized structures. In addition, redox catalysis allowed to support chemical reactions that would not otherwise have taken place. This complexification in form and function led to a multiplication of reactions and possible interactions that may have been an important evolutionary driver for biological diversification and the development of specialized physiological functions in complex organisms (Figure 4A(Fig. 4); Reference in Figure 4: Santolini et al., 2019[54]). All these redox structures gave rise to many parallel redox processes that proceed simultaneously at distinct levels of biological organization, mutually embedded into one another, from individual protein up to the ecosystem. As this architecture evolved and became richer with time, its individual redox structures remained embedded within a unique redox system (the '*redox spine'*) that allowed keeping all metabolic activities fully synchronized across the whole organism (Figure 4B(Fig. 4)). These considerations lead to the idea that electron exchange processes within unicellular and multicellular organisms (particularly for coordinated biological development in metazoans) must be intimately linked to fundamental origin-of-life chemistry (Santolini et al., 2019[54]).

## What is Life? Fundamental Principles of Operation

All life forms are essentially open systems because they interact with their wider environment (Von Bertalanffy, 1968[72]); their special characteristic is that they are defined by a space within a confining boundary. Thus, there is the need to differentiate the space within a boundary, identified as the '*internal milieu'*, from the wider external environment, the '*external milieu'*. The interactions between the internal milieu and the external milieu are selective. Everything contained within the space identified as the internal milieu is highly structured and enables organized function. To achieve and maintain the structural and functional organization of e.g. an organelle, a cell, or an organ demands energy and can be considered to represent '*negative entropy*'. The associated chemical reactions that enable this state operate far from equilibrium thereby countering the fundamental second law of thermodynamics towards entropy. To achieve this requires a continuous flow of energy equivalents that support a state of dynamic equilibrium and turnover of all constituent parts.

The organization and function that characterize higher forms of life can be considered as having different levels of complexity from the molecular, through sub-cellular to cellular, tissue and organ up to the level of the whole body. Harmonization across the different levels of organization requires integration and regulation. Given that the nature of the boundary-limited space is characterized by negative entropy, there is the need for an ongoing exchange with the environment to access the energy equivalents and chemical building blocks required to maintain structure, function and a dynamic equilibrium within and between levels of organization. 

Ongoing exchange with the environment is therefore an intrinsic feature of living systems and required to achieve some form of net acquisition during replication or growth or maintenance of the quasi-steady-state characteristic of adulthood (in humans) over any extended period of time. For higher organisms, any general framework has to account for a multilevel landscape that has emerged through complexification in a self-organizing fashion (Bak, 2013[2]). We have explored the extent to which electron exchange (redox) reactions as emergent intrinsic feature of evolution can adequately account for enabling the acquisition of specific functions (Santolini et al., 2019[54]). 

There is another form of interaction between the internal and external environments, in which the external environment is seen as potentially hostile to the integrity of the structure and function of the internal environment. The potential advantage conferred by a regulated internal environment enables more refined chemical behaviors, but this has to be set against the need for any organism to be able to cope with the adverse effects of a potentially damaging environment. 

The structures and functions that we observe today are presumed to represent those that have best enabled survival within the environments encountered during successive past periods of evolutionary development, i.e. the response to novel challenges has to be seen as adding to or building upon past experiences. 

The common consideration of ill-health or pathology still tends to focus on the manifestations of adverse consequences, which may express themselves differentially at any level of organization. Yet, taking into account the principles and processes described above enables characterizing “health” as an active process and understand how this status may best be achieved and sustained. The details and specifics of these emergent properties have been explored extensively in the literature. Our concern here is to consider how these might operate within and across different levels of organization.

We have cited the work of Selye and Sies as indicative of the perspectives that can be drawn from studies carried out at different levels of organization. Together, they are illustrative of many elegant presentations that have been made across a wide swathe of systems biology applied in different contexts. Each area of enquiry tends to develop its own model(s) of how the system might operate at that particular level of organization. It is often less obvious how the different levels of organization might interact effectively. Applying understanding across different levels of organization is not straightforward. In part, this may be attributed to the particular underlying considerations that determine the framework adopted to explore specific processes at particular levels of organization; these are often implicit and assumed within a particular field of interest. In order to facilitate movement between seeming widely different areas of interest it is necessary to be explicit about the underlying considerations of importance to facilitate communication and share experience. 

We have identified five principles we consider to be fundamental to the characterization of an integrated stress response that might operate across different levels of organization and for the body as a whole:

**1.** Living organisms are characterized by having a boundary, which for humans is most obviously the skin and the mucous linings of the gastrointestinal, respiratory and urinary tracts. The boundary identifies and differentiates self from non-self, the internal milieu from the external milieu. Within these boundaries are sub-compartments, subcellular, cellular, tissue or organ with integrated behavior within and across each level of organization and inter-relating hierarchically within the whole body. A characteristic that is evident both within and between levels of organization is that there is both specialization and cooperation.

**2.** An important feature within the boundary is the tendency to maintain a stable internal environment while still being connected to and allowing communication with the external environment. This capability for homeostasis is a dynamic feature of health. It is self-regulating, characterized by defined structures, compositions and function, and an ongoing external exchange with the environment in the form of air (and volatile compounds), water and nutrients. 

**3.** The external environment may impose challenges or stresses. The ability of the internal environment to cope with or protect its integrity against these challenges is structured and organized. The level of the response appears fit for purpose with the ability to recruit more extensive, integrated changes according to the hierarchical relationships and in relation to the magnitude of the challenge.

**4.** Following exposure there is the ability to acquire learned responses to the challenges or stressors with the opportunity for the response to become more effective so it is more readily accommodated on repeated exposure. For milder forms of challenges this response appears as the ability of the organism to adapt, which presents as variation in non-specific behavior. With more intense or specific stressors (such as microbial infections or radiation-related damage, for example) it may require a greater coping efficiency.

**5.** In higher organisms, these responses appear to be increasingly complex. It is assumed that these characteristics are acquired progressively as the product of evolutionary experience and manifest as the characteristics of emergent properties within hierarchical systems. In this sense the response to any novel challenge or stressor builds on earlier experience and the response adds to the existing repertoire of capabilities. Therefore, any “cross-sectional” characterization of responsive behavior across stressors and species recapitulates a longer experience over time, and might only be understood against the background of this progressively acquired experience. 

Thus, for human biology each discrete observation has to be related to the wider whole and interpreted as one component of a more complex, multidimensional experience. In order to better organize our thoughts we developed the concept of the RSI. With time, this concept has matured and the description below reflects our current understanding.

## Characteristics and Evolutionary Origin of the Reactive Species Interactome

We originally defined the RSI as a redox system of chemical interactions of reactive species such as RSS, RNS and ROS with themselves and with downstream biological targets (Cortese-Krott et al., 2017[8]). In addition to these reactive species we consider that it may include other small chemicals (Fukuto et al., 2012[21]) that are often referred to as '*gaso-transmitters*' (including NO, H_2_S, CO, COS) (Steiger et al., 2018[66]; Wang, 2003[74]) as well as hydrogen (H_2_) and ammonia (NH_3_). It represents a dynamic biochemical interface that connects the internal milieu with the external environment and allows cells or whole organisms to accommodate changes in metabolic demand or environmental conditions (Cortese-Krott et al., 2020[9]) (Figure 2(Fig. 2)). The principal reaction partners of reactive species (which became primary '*biological targets*' in the course of evolution) are metal centers and protein thiols. The reaction with protein thiols leads to a variety of post-translational modifications, which may induce structural and/or functional changes. The outcome of these reactions will depend on the function of the biological target (i.e. whether it is an enzyme, a transcription factor, an ion channel, or a critical regulatory element within a signaling pathway) and the nature of the modification and may result in short-term (e.g. by post-translational modification) or long-term effects (via regulation of gene expression). 

The RSI is characterized by i) the ability to sense the environment, transduce and translate signals into changes in cellular activity/function; ii) versatility and specificity; and iii) rapid response and robustness. These characteristics can be understood considering the following:

**i)** *Ability to sense the environment and transduce/translate signals into changes in cellular activity or function*. As a system that functions by enzymatically transforming precursors (sulfur-containing amino acids, arginine and inorganic substrates such as oxygen, nitrite, and sulfate) into small reactive molecules the RSI has the ability to sense changes in environmental conditions. With parallel generation of multiple reactive species at controlled rates the mixture of reaction products arising will vary as a function of even minute changes in conditions. These changes are sensed by an array of cellular receptors with overlapping chemical specificity; coupled to a suitable transduction mechanism, those changes are then translated into a biological response. The sensing mechanism used by the RSI is thus not dissimilar to the olfactory response (or the principle of an electronic nose); however, instead of relying on receptors alone, it uses a regulated chemical sensing process that enables the cell to actively “sniff out” its environment. The nature of the chemical process itself resembles the principle of SIFT-MS, a mass spectrometry based technique for the detection of volatile species at trace level. The ability of the RSI to induce changes in enzymatic activity (e.g. by post-translational protein modifications or regulation of gene expression) allows signals to be transduced by inhibiting or activating multiple downstream effector pathways; collectively, this enables cellular adaptation to better accommodate new conditions/demands.

**ii**) *Versatility and specificity*. By virtue of its integration with the cellular oxidant/antioxidant network the RSI enables living systems to integrate and synchronize energy generation and expenditure, intermediary metabolism and mitochondrial function. In multicellular organisms, this requires information as well as substrate and product exchange across levels of biological organization and between cells/tissues with highly specialized functions in a coordinated fashion. In this regard the extracellular fluid (lymph and blood) can be considered to function as both '*communication highway*' and '*trade route*', providing the opportunity for local redox-dependent processes to achieve synchronization with all other electron-exchange processes at the whole-body level.

Rapid/dramatic changes in metabolic demand or environmental conditions may represent challenges/threats that place a demand on energy provision in order to sustain Life. This requires an active sensing/transduction/adaptation process that is versatile, fast and robust. Importantly, the emergent system must be able to respond to novel conditions and chemicals. We suggest that this is achieved through a regulated, active sensing process that utilizes simple chemicals with moderate-to-high reactivity. Examples include entities such as hydrogen sulfide (H_2_S), NO and superoxide (O_2_^.-^)/hydrogen peroxide (H_2_O_2_) all of which can be produced biologically at defined rates using specific enzyme systems. These primary products of the RSI are continuously generated, at defined rates, by enzymatic reactions. The main chemical interactions of the RSI with their biological targets include one- and two-electron oxidation and reduction steps, nitrosation, nitration, sulfuration, and polysulfidation reactions, complemented by radical-radical combination and addition reactions. 

Each RSI constituent and reaction product has a specific reactivity and physicochemical property (Fukuto et al., 2012[21]), which define their travel distance and lifetime (and thus action radius) in the biological environment (Cooper et al., 2002[7]; Hill et al., 2010[26]; Winterbourn, 2008[77]). The chemical space covered by the richness of the reaction products formed allows sensing of known and unknown chemical entities over a wide range of reaction conditions (temperature, osmolarity, pH, pO_2_), (see Travasso et al., 2017[70]). As with other complex systems under kinetic control, this has the potential for almost infinite possibilities of chemical interactions between these reactive species with chemicals in the external environment and biological targets in the internal environment. However, the structured internal environment places limits on these options, enabling biology to exploit only a subset of possible reaction channels to achieve a particular function (Travasso et al., 2017[70]).

**iii)** *Rapid response and robustness.* The speed and promiscuity of reactions between the different constituents of the RSI leads to almost instantaneous changes in the pattern of reaction products formed and allows cells, via changes in downstream effector signaling, to rapidly respond to alterations in metabolic demand (depending on the availabilities of precursors and reducing cofactors) and environmental conditions (substrate availability). The RSI is highly robust because multiple system of in-built redundancy in enzymatic and non-enzymatic reactions that either produce or consume the reactive species and because of the chemical richness of reaction products simultaneously generated. Near instantaneous fine-tuning is enabled by subtle changes in fluxes of reaction products, giving rise to different product mixtures at any one time and condition. 

The multidimensional nature of the RSI thus confers the characteristics of a 'smart membrane' to cellular boundaries enabling them to register changes in both, nature and composition of the external environment while comparing the appropriateness of the internal metabolic status with previous exposure experiences to best execute the physiological function it emerged to fulfil. In addition, the evolutionary process confers “learning” and anticipatory capabilities since evolutionary iterations that are 'unfit for purpose' under new environmental conditions simply vanish. Taken together, these characteristics allow the RSI to fulfil an integrative function of sensing multiple stressors, allowing to adjust bioenergetic/metabolic needs and activate downstream effector pathways accordingly to ensure the organism stays fit for purpose.

The evolutionary origin of the RSI must have preceded the '*Great Oxidation Event'*. We and others (Olson and Straub, 2016[49]; Cortese-Krott et al., 2017[8]) have argued that the fundamental building blocks and regulatory elements of Life used by all contemporary cells and organisms evolved long before the emergence of oxygenic photosynthesis in cyanobacteria, which marked the transition from a reducing to an oxidizing environment. In fact, 7/8 of the development of Life on Earth occurred in an anoxic environment rich in H_2_S and iron sulfides (Wächtershäuser, 1992[73]; Olson and Straub, 2016[49]), possibly explaining why sulfur plays such a fundamental role in supporting Life. The accumulation of oxygen in the atmosphere and the change to an aerobic world may have been the key driver for the development of eukaryotes, the result of a symbiotic relationship between a small aerobe and a larger anaerobe, with the former becoming the oxygen-fuelled power plant we today recognize as the mitochondrion (Lane, 2006[35]). 

While the use of oxygen as terminal electron acceptor enabled a far more efficient production of molecules that can be used by cells to generate energy at the same time it forced life forms to deal with oxidative by-products and run some of the redox processes optimized for use in a reducing environment in reverse. This view is supported by the finding that enzymes such as catalase and superoxide dismutase emerged well before a need arose to detoxify ROS, and the same proteins can oxidize H_2_S to polysulfides (Olson et al., 2017[48], 2018[47]). Thus, it is possible that these *antioxidant* *enzymes* were first used by Nature to handle RSS rather than inactivate ROS (of note, per- and polsulfides have both pro-oxidative and antioxidant properties (Fukuto et al., 2018[22])). This highlights the need to carefully consider the evolutionary history of physiological processes, when taking a cross-sectional view onto regulatory elements of intermediary metabolism in contemporary organisms. In any case, the sensing and adaptation capabilities of the RSI were greatly enhanced by the additional inclusion of ROS (following the utilization of RSS and RNS) as this increased the number of chemical interaction products. 

Consideration of these evolutionary developments suggests that sulfur chemistry represents the common thread upon which all later capabilities were built and invites the articulation of a sixth principle of operation (see section “What is Life? Fundamental principles of operation”) around the use of sulfur by living systems:

**6.** Sulfur sits at the heart of the “life support” system that enables cells to harness the opportunities offered by this particular element in the form of inorganic (H_2_S, sulfate) and organic (cysteine, glutathione) redox molecules for communication as well as metabolic and energetic regulation. Thus, sulfur utilization has enabled lateral and vertical integration of the different levels of organization, both for the complex intra- and inter-cellular communications required for growth and development and the systems through which the constancy of the internal milieu can be maintained in the face of external stressors.

The primordial reductant H_2_S would have been too reactive and too mobile for it to be used effectively within compartmentalized cell structures. This was resolved by transformation of inorganic sulfur into organic molecules (via conversion of serine into cysteine). In proteins, the incorporation of this amino acid not only offered enhanced stability in more complex structures like peptides and proteins (via formation of structural disulfide bridges), but its sulfhydryl group could also be utilized as a redox switch and reactive moiety to fulfil enzymatic functions (functional -SH). Incorporation of cysteine into the tripeptide GSH enabled this highly reactive amino acid to be held in a cellular form that supported more complicated regulation of responses. Yet, in the presence of oxygen, one major challenge remained: free thiols would quickly become oxidized to disulfides and sulfinic/sulfonic acids by e.g. H_2_O_2_, requiring reducing systems to ensure their secure function.

## The Significance of Nitric Oxide, Reducing Power and the Transsulfuration Pathway

Adequate formation and availability of NO is thought to play an essential role in protecting the sulfur-based metabolic and redox signaling circuitry from overoxidation (Cumpstey et al., 2021[10]). Formation of NO by the NO synthase family of enzymes requires the availability of molecular oxygen and NADPH for reduction of the intermediate N-hydroxy-L-arginine (Stuehr and Haque, 2019[68]). However, NO can also be formed via the non-canonical pathway of the nitrate-nitrite-NO reduction pathway (Lundberg et al., 2009[38]), and it is conceivable that NO may have been generated biologically via a reductive pathway well before the emergence of oxygen in the atmosphere (Feelisch and Martin, 1995[16]). In an aerobic world, NO has long been known to act as a chain-breaking antioxidant to prevent lipid oxidation (Wink et al., 2001[76]), but its role in protecting the critical elements of stress signaling (elements that evolved in a sulfur-rich world essentially free of oxygen) does not appear to have been recognized before the onset of the current pandemic. Under both, reducing and oxidizing conditions NO would react with thiyl radicals (RS**^.^**) much more rapidly than oxygen, giving rise to S-nitrosothiols (RSNO). These species are resistant to oxidation and can be enzymatically recycled to the free thiol form with the aid of reducing enzymes (denitrosylases; e.g. Stomberski et al., 2019[67]).

In an aerobic world, organisms rely on the availability of sufficient '*reducing powe*r' (i.e. the propensity to donate an electron from one compound to another) to sustain Life. This reducing power is generally provided by the generation of NADH and NADPH following oxidation of organic molecules (e.g. glucose in glycolysis, the tricarboxylic acid cycle and the pentose phosphate pathway). The major universal electron recipient in these processes is oxidized nicotinamide adenine dinucleotide phosphate (NADP^+^), which is thereby converted into its reduced form (NADPH) and subsequently used by cellular NADPH-dependent enzyme systems to reduce inorganic or organic substrates. A major proportion of NADPH is used for anabolic reduction processes and consumed by thioredoxin reductase (TrxR) and glutathione reductase (GR), which sequentially convert disulfides (thioredoxin-disulfide and GSSG, respectively) into their reduced forms. By comparison, the NADH/NAD^+^ redox couple is used mostly for catabolic reactions, e.g. to extract energy from organic molecules.

In addition to providing reducing power for DNA synthesis, NADPH-dependent TrxR and GR support a significant part of the antioxidative enzyme machinery with the cytosol kept in a balanced reduced state, preventing oxidative stress and repairing oxidative damage (methionine sulfoxide reductase, peroxi-redoxins, glutathione peroxidase). Interestingly, deletion of either the TrxR or GR system does not substantially alter cellular redox status or GSH concentration. However, consistent with its emergence coinciding with metazoan development, disruption of TrxR has profound effects on embryonic viability and cell signaling, leading to a disruption of embryonic patterning and Nrf2 regulation (Dagnell et al., 2018[12]). Thus, not all disulfide reducing systems serve solely antioxidant roles and/or are mutually compensatory; instead, they often cater to rather specific reaction channels and are not readily replaceable by other reducing equivalents.

Eukaryotic cells have an additional NAD(P)H-independent, methionine-dependent system that donates electrons when the former systems are unavailable or compromised (Miller et al., 2018[43]). This system was unmasked after development of a murine model in which all hepatocytes were constitutively devoid of both, TrxR and GR. Surprisingly, these mice were not only long-term viable and displayed relatively normal liver function, they were also resistant against oxidative stress induced hepatic ischemia/reperfusion damage. In a series of elegant experiments it was shown that the activity that sustained redox homeostasis in the complete absence of constitutive disulfide reductase activity was linked to the *de novo* synthesis of cysteine via the methionine recycling/transsulfuration pathway to allow high fluxes of reduced glutathione (GSH) to be synthesized. To synthesize GSH from methionine, equimolar amounts of glycine, glutamine and serine are required. For humans and other metazoans, methionine is an essential amino acid. In addition to serving as a source of reducing power to maintain redox homeostasis it is also required to provide S-adenosylmethionine (for methylation reactions), coenzyme A (for fatty acid processing through the TCA cycle), taurine (as an osmolyte and for conjugation to bile acids), and H_2_S (for signaling purposes). 

The central role of glutathione for cellular and whole-body function is a pertinent example suited to illustrate the complexity of redox regulation in the context of the six organizing principles described above, allowing operational integration with other bodily systems and alignment of the stress concepts discussed by Sies and Selye.

## Glutathione – From Mitochondria to the Whole Organism

Glutathione is a tripeptide comprising glutamate, cysteine and glycine (γ-Glu-Cys-Gly). For a long time, these three amino acids were thought to be non-essential, but cysteine and glycine were later shown to be conditionally essential (Jackson, 1991[31]). Glutathione is exclusively produced in the cytosol of most cells from where it is distributed to cell organelles including the nucleus, the endoplasmic reticulum and the mitochondria. At the whole-body level, the majority of glutathione is produced in the hepatocytes of the liver; following excretion it is transported, through the blood, to other cells and peripheral tissues (Meister and Anderson, 1983[42]; Griffith, 1999[24]; Lu, 2013[37]; Sies and Wendel, 2012[64]). Production and oxidation as well as reduction and metabolism of glutathione are controlled by different enzyme systems, which allow discrete control of the forward and backward reactions, consistent with the view that GSH represents an important check point in intermediary metabolism (Newsholme, 1970[44]). If indeed GSH played a fundamental role in cellular redox regulation beyond the mere protection of cells from the adverse effects of damaging free radicals, it would make sense that its concentrations were well protected using multiple checks and balances. Effective intracellular GSH concentrations are maintained by several cofactors and metabolites, the activity of which depends upon the availability of specific vitamins and minerals. Examples include selenium for glutathione peroxidase as well as riboflavin and NADPH for glutathione reductase activity, whereas NADP^+^ itself is formed from nicotinic acid with the reducing power of NADPH derived through the hexose monophosphate shunt (which requires glucose and thiamine). That these interrelationships also operate in the opposite direction is exemplified by the GSH-mediated protection of vitamin B_12_ from depletion by reactive xenobiotic metabolites (Watson et al., 2004[75]).

In conjunction with ascorbate and alpha-tocopherol, reduced glutathione (GSH) acts as a major intracellular antioxidant. Oxidized glutathione (GSSG) is either exported from the cell (which is compensated for by *de novo* synthesis of GSH) or reduced back to GSH at the expense of NADPH. The GSH/GSSG ratio is frequently used as a measure of intracellular redox status. GSH also forms mixed disulfides with protein thiols (S-glutathionylation), is involved in the regeneration of protein S-nitrosothiols (Clementi et al., 1998[6]) and can react with cysteine persulfide and other polysulfides via transsulfuration reactions (Ida et al., 2014[29]). Serum albumin (in humans, with a single reactive -SH group in Cys-34) serves as the major extracellular antioxidant in larger organisms, and represents an important transport form for other low-molecular-weight thiols (Cortese-Krott et al., 2017[8]). Extracellular glutathione concentrations are very low, and recent studies demonstrated that the cysteine/cystine couple in human plasma is not in equilibrium with the GSH/GSSG redox couple. This suggests independent levels of regulation, which may be used diagnostically to assess the risk of e.g. cardiovascular disease (Patel et al., 2016[50]).

But GSH is much more than an antioxidant. Via the gamma-glutamyl cycle it is also a source of cysteine. In addition, it serves as a cofactor for enzymes involved in the conjugation and detoxification of xenobiotics (glutathione S-transferase), the removal of lipid peroxides (glutathione peroxidase), and the production of nitric oxide (nitric oxide synthase); a number of other cellular functions including amino acid transport, protein structure and synthesis, prostaglandin/leukotriene signaling, calcium homeostasis, cell signaling and autophagy as well as mitochondrial function are either associated with or dependent on a normal glutathione status (Jackson, 1990;[30] Flohe, 2019[17]; Aquilano et al., 2014[1]). Inhibition of whole-body glutathione synthesis induces oxidative stress and leads to severe hypertension, possibly caused by an impaired production of NO (Vaziri et al., 2000[71]). Likewise, systemic inhibition of NO synthesis (which causes hypertension and oxidative stress in its own right) is associated with a perturbation in glutathione synthesis (Pechánová et al., 1999[51]), demonstrating the interrelatedness and possible interdependency of these two “antioxidants”.

In the absence of intentional pharmacological intervention, a fall in glutathione concentration implies a limited ability for its synthesis due to the inadequate availability of the precursor amino acids, a decreased availability of ATP, limited availability of cofactors, or an accelerated utilization/consumption, loss or breakdown. Under unstressed conditions this does not necessarily translate into a different redox status (represented by the GSH/GSSG ratio) (Diederich et al., 2018[13]); however, it does represent a limitation of the buffering capacity of cells/tissues when required to deal with a further oxidant insult (decreased antioxidant reserve capacity (Diederich et al., 2018[13]; Erkens et al., 2018[14])). Accelerated GSH utilization due to e.g. chronic alcohol consumption or frequent use of over-the-counter medications such as paracetamol can render the human body more susceptible to additional stressors such as viral infections. The resultant impaired ability to cope with competing demands may explain those individuals' greater susceptibility to poor clinical outcome from COVID-19 (Feelisch et al., 2021[15]). The crosstalk between glutathione and NO production may underpin the increases in blood pressure associated with chronic paracetamol use (MacIntyre et al., 2022[39]).

Importantly, the demand for any particular pattern of amino acids varies and the relative need for specific amino acids can be increased in situations such as malnutrition, diarrhea and inflammatory gastrointestinal failure where there are increased unbalanced losses. As a result, the imbalance between availability and metabolic demands of the body can create a functional deficiency state that is not easily remedied by provision of the pattern found in usual diets. The same holds true for generic multivitamin supplements where the pattern of demand for specific minerals or cofactors may be changed significantly in disease states. Similar concepts have been developed in relation to the requirements of L-arginine and glutamine for tissue repair and immune function (Barbul, 1986[3]; Newsholme et al., 2003[45]) and COVID-19 (Cumpstey et al., 2021[10]). Aging, diabetes, pulmonary or hepatic fibrosis and alcoholic liver injuries have also been associated with a dysregulated glutathione synthesis (Griffith, 1999[24]; Lu, 2013[37]). Based on the discussion above, this may be interpreted as resulting in a constraint capability to generate sufficient reducing power at times when demand is increased.

Important considerations that remain unresolved include the compartmentalization of redox systems between different cell organelles, cells, and tissues; the principle of harmonization of extracellular and intracellular redox status; and the assurance of suitable redox zones for optimal operation of different cell organelles and cell types; (see e.g. Selvaggio et al., 2018[57]; Travasso et al., 2017[70]). For example, the endoplasmic reticulum requires a more oxidized redox environment to enable proper protein folding compared to the cytosol, whereas mitochondria are more reduced, with considerable heterogeneity between mitochondrial intermembrane space and matrix. 

Mitochondrial glutathione plays an important role in maintaining and defending the redox environment that is most appropriate to fulfil its bioenergetic and highly specialized metabolic functions (Mari et al., 2009[40]). In addition to preventing structural ROS-induced damage mitochondrial glutathione exerts influence over lipid (cardiolipin) oxidation, programmed cell death (apoptosis), and membrane permeabilization. A number of pathologies and aging are associated with progressive mitochondrial dysfunction and reduced glutathione content (Ribas et al., 2014[53]). In most cases, the reason for the dysfunction remains unknown, although perturbations in facilitated glutathione transport across the mitochondrial inner membrane, chronically elevated mitochondrial ROS production with alterations in electron flow along the mitochondrial respiratory chain, and lipid peroxide-induced alterations in Nrf2/Keap1 sensing are likely candidate mechanisms. 

Among the many different approaches that have increasing life span and stress resilience as an objective, long-term dietary restriction has shown most consistent benefits across species. The evolutionary conservation of the transsulfuration pathway (allowing for the production of H_2_S) appears to play a critical role in the dietary restriction-related benefits on stress resistance (Hine et al., 2015[27]). Moreover, stimulation of the hypothalamic-pituitary axis has been directly implicated in the regulation of H_2_S production (Hine et al., 2017[28]): the part of stress signaling that sits at the heart of the responses characterized in Selye's '*general adaptation syndrome*'.

The release of glucocorticoids from the adrenal glands in response to activation of the HPA axis is known to be associated with oxidative stress (Spiers et al., 2015[65]). Glucocorticoids stimulate mitochondrial energetics and ROS generation, and this cross-talk appears to modulate immune responses and determine the clinical manifestations of patients with sepsis (Kasahara and Inoue, 2015[34]). However, untargeted antioxidant therapy (with N-acetylcysteine or vitamin E) does not resolve these oxidative stress related perturbations; instead, it induces HPA axis hyperactivation (Prevatto et al., 2017[52]), illustrating the dangers of not giving adequate consideration to the complexities of oxidative stress-related problems such as the need to adequately allow for dose-response relationships within non-linear systems (Travasso et al., 2017[70]; Selvaggio et al., 2018[57]). It is conceivable, that chronically elevated glucocorticoid concentrations lead to mitochondrial glutathione depletion; if this was accompanied by an impairment in mitochondrial glutathione handling and/or H_2_S-related signaling under control of the thioredoxin system this would explain why generic antioxidant supplementation does not provide any benefit in this situation.

## The Reactive Species Interactome in the Context of Stratified Medicine

The ideas of 'patient stratification' and 'personalized medicine' have come to be used in a clinical context to acknowledge biological variability (Cortese-Krott et al., 2017[8], 2020[9]). These terms are often used interchangeably along with 'precision medicine', which purports to provide a specific and individualized therapy on the basis of a global analysis of the genetic and metabolic status of an individual patient. Although the technical developments related to carrying out omics approaches and the analysis of 'big data' are advancing rapidly considerable knowledge gaps remain, preventing its implementation into routine clinical practice. Current practice is considerably less sophisticated, focusing on the measurement of vital signs, biomarkers of organ damage and inflammation and often treating 'signs and symptoms' rather than tackling the causes of diseases. 

Being fashionable, the above terminology has been applied in a wide range of situations without any consistency and sometimes with a lack of clarity. The RSI offers a structured framework in which the ideas around these important considerations might be better organized, and by enabling seemingly unrelated threads at different levels of hierarchical organization to be related to one another, permitting more productive investigation. As a normal feature of clinical diagnosis and care, patients are stratified by disease entity, and this is usually applied to the exposure considered to be causative; often, this is related to a challenge from the external environment: infection, toxin, or trauma, for example. In this context there may be stratification in terms of the degree, timing and intensity of the relevant stressor. 

The more recent focus on the context within which the response to stressors takes place implicitly relates to the host's responsiveness. In this context, it is important to consider from primary organ/tissue the response emerges, to have a sense of the hierarchy and levels of response that might be recruited, and the factors that contribute to that response (endotype). It acknowledges the contribution to variability of defined factors such as age, gender and genotype, but has some difficulty in allowing for “learnt experiences” as, for example, in relation to epigenetic phenotype or acquired memory associated with specific immune responses to infection. Furthermore, within specific disease processes, the stage of the process (which can be considered as a dynamic and changing interaction between the stressor and the host response) has not been adequately addressed.

The field of redox metabolomics (i.e. a global interrogation of cellular redox physiology at the whole-body level) is still in its infancy. Part of this is due to the unstable nature of many redox entities and the multitude of interactions in which they are engaged. Moreover, many highly active biomolecules are found only at very low concentrations in blood cells and biofluids, and many redox-related metabolites are not part of the routine panel of metabolites covered to enable precision medicine. 

We recently suggested a way forward to disentangle the redox architecture (Cortese-Krott et al., 2017[8]) by applying an 'omics approach that is targeted at critical elements of the RSI, including (1) the precursors/substrates (O_2_, amino acids, cofactors involved in redox control), (2) the transducing elements (cysteine-based redox relays and the dynamic interplay of circulating reduced and oxidized as well as protein-bound thiols), and (3) the stable end products of the RSI (S/N/O-based metabolites). If such measurements were conducted in a systematic manner alongside the routine clinical/blood phenotyping, perhaps complemented by the measurement of compounds central to metabolic regulation and mitochondrial function, it should be possible to disentangle the network structure and modes of interactions. One example of such integrative markers of metabolism is homocysteine, which represents a combined read-out of methionine and vitamin B_6 _and B_12_ availability, one-carbon metabolism (methionine recycling and tetrahydrofolate pathways), and flux through the trans-sulfuration pathway. 

Other important considerations relate to the flux of metabolites through a particular pathway as well as the concentration of a single substance at any particular time, demanding the analysis of metabolic fluxes through redox pathways. This consideration alone suggests that we should aim at collecting longitudinal metabolic data (e.g. by taking samples at different time points from the same individual) rather than random snapshots of a highly dynamic system. At the very minimum, we ought to capture the dynamics of regulation, and steady-state levels at baseline should be compared to levels upon systematic perturbation of the whole system (e.g. sedentary/exercise, normoxia/hypoxia, starved/fed, etc.). Such a systemic dynamic approach may help to identify new pharmacological targets of redox signaling, as well help the risk stratification of patients, improve recovery from critical illness or major trauma and inform nutritional priorities for healthy aging.

A recent feasibility study in a small number of healthy individuals exposed to combined environmental and metabolic stressors (a moderate bicycle exercise under normoxic and hypoxic conditions), revealed a wide difference in the pattern of change in arterio-venous plasma concentrations of redox metabolites even though the challenges were identical (Cumpstey et al., 2019[11]). Another remarkable feature was the dynamic nature of the alterations upon onset and cessation of the acute stressor (physical activity) superimposed onto the chronic stressor (hypobaric hypoxia). These observations are consistent with the notion that the redox readouts assessed respond rapidly to acute alterations in metabolic demand.

These observations in healthy individuals suggest that quantification of a similar panel of redox metabolites in disease may add value to biomarker/clinical measurements performed as part of routine clinical care of patients or in dedicated research studies. The dynamic nature of these readouts implies that it will be important to faithfully capture the temporal pattern of redox metabolome changes in biofluid concentrations to get a handle on the trajectory of the disease process under study. This will add an additional dimension (time) to any cross-sectional observation of the self-organizing, complex multi-layered regulatory network that underpins physiological function (Santolini et al., 2019[54]). Importantly, it will allow a determination of where the patient currently is on their health/ill-health journey and the likely direction of travel (recovery/deterioration). Indeed, models of such approaches have demonstrated hysteresis loops that “map the disease space” and offer the potential for tracking resilience to infections or more generally for tracing differences in health curves between individuals (Schneider, 2011[56]; Torres et al., 2016[69]), with potential application to assessing comorbidities in population-based studies (Giannoula et al., 2018[23]). 

This sort of analysis should be eminently applicable to the field of redox systems biology and complement kinetic modeling studies providing novel insights into how the biological environment constraints chemical reactivity and enables oxidant signaling (Selvaggio et al., 2018[57]). Meanwhile, we ought to provide a solid basis for future systems modeling by generating high-quality multi-omic datasets that capture the spatio-temporal dynamics of key read-outs of the RSI in parallel with tight physiological phenotyping. Ideally, such data should be obtained from experiments carried out in healthy human individuals subjected to usual physiological variability as well as to defined stressors and degree of stress (e.g. exercise vs. rest, hypoxia vs. normoxia, heat vs. cold, fasting vs. controlled feeding), with synchronous sampling from multiple compartments (e.g., plasma and blood cells, saliva, urine, exhaled breath). Using multi-level statistical modeling/analysis methods in combination with topographical data analysis (a combined analysis/visualization technique that preserves the shape of data rather than reducing its dimensions) it should be possible to capture specific perturbations of the whole-body redox network in response to stressors.

## Summary and Conclusions

Redox Biology plays a fundamental role in understanding how living systems work, and oxidative stress appears to be either associated with certain diseases or represents an etio-pathological factor in its own right. To make progress in this field of research requires the development of a conceptual framework capturing the whole complexity of chemical interactions within model organisms and real biophysical and biochemical systems. We posit that the production and interaction of reactive oxygen, nitrogen and sulfur species are essential to adaptive changes, thereby enhancing the opportunity for survival, with sulfur playing a central role because of its involvement in the early stages of development of Life on Earth. At that stage, small molecules (gases, metals, S/N/O-based compounds) became intimately involved in multiple redox interactions, forming a bridge between inanimate and living matter. Thus, reactive species are constituents of an integrated redox signaling network we defined as *Reactive Species Interactome*. This system is used by all contemporary organisms to sense and accommodate environmental stressors. Biological systems have to be considered as a whole and in relationship with their environment. Constituents of this system can be studied in isolation, but as all are intimately intertwined, both functionally and chemically, with multiple other signaling and transduction pathways, intermediary metabolism and mitochondrial function, at some stage these inter-relationships across and between different levels of organization have to be brought together. 

Electron exchange (redox) processes provide the basis for the synchronization of the entire redox network across levels of biological organization by acting both as* lingua franca* and mechanism of this chemical interaction. In addition, all elements of this *Redox Interactome* have multiple modes of action. Being able to reliably capture multiple facets of the RSI across different compartments in a time-resolved manner will be fundamental to understanding the principles of regulation in Redox Biology. Combining this with mathematical modeling approaches that represent disease trajectories has the potential to guide therapeutic approaches in the emerging field of Redox Medicine and may also aid in assessing nutritional needs, individual risks for certain interventions and develop therapeutic strategies. 

Sulfur's versatile redox chemistry is further enriched by complex interaction with reactive nitrogen (e.g. NO, HNO) and reactive oxygen species (e.g. O_2_^.-^, H_2_O_2_). While NO may have been the first gaseous molecule that was harnessed for inter-cellular communication and antioxidant protection, triggering the formation of symbiotic relationships (Feelisch and Martin, 1995[16]), sulfur would appear to sit at the core of the *Redox Code*, enabling to exploit the opportunities for more efficient energy provision and coordinated development of specialized function in multicellular organisms. Understanding how this intricate system operates to promote stress resilience while maintaining whole-body redox balance will be important to advance redox medicine and modulate electron exchange processes to sustain health, avoid development of ill-health and treat overt disease.

## Declaration

### Acknowledgment

No specific funding was received for the production of this article.

### Conflict of interest statement

MF is a consultant for ASEA Global; all other authors declare that they have no conflict of interest.
